# On the County Hospitals of Ireland

**Published:** 1806-04

**Authors:** 


### *To the Editors of the Medical and Physical Journal.*

GENTLEMEN,

IN the Number of your Journal, for the present month, I have just read a paper, the writer of which professes to suggest "Means of supplying the poor more effectually with medical assistance in the country parts of Ireland." As the writer of that paper, if we are to judge from its contents, is defective in the knowledge of the subject on which he writes, I request a place in your Journal for a few facts and observations, that his errors may be corrected, and that the minds of those of your readers, to whom the right sources of information are not open or accessible, may not be misled by incorrect representations.

The author of that paper, who assumes the *virtuous* signature of ANTIPECULATOR, begins with saying, "that, in order to supply receptacles for the sick poor, in Ireland, the legislature voted several considerable funds for the erection and endowment of hospitals, to be provided in the most central and populous town of each county."—Whereas, the fact is, that the whole fund then voted for each hospital, amounted to no more than 200l. *per annum,* one half to be issued from the national treasury, and the other half to be raised by grand jury presentment, on the county at large. Afterwards, when it was determined to annex to each hospital, cells and apartments for lunatics and ideots, a law was passed, authorising grand juries to make additional provision for the maintenance of those deplorable examples of human infirmity; but as the sum for each is only 4l. a year, exclusive of what may be necessary for cells and clothing, and the number in each hospital rarely exceeding fifteen, there is reason to fix the *maximum* expense at 50l. diminishing, in different years, until it go as low as 30l. *per annum.* So that, at the utmost, the funds, which the author terms *considerable,* did not exceed 250l. a year. But as the majority of the counties have not availed themselves of the provision made by the law for lunatics and ideots; it follows that, instead of the *considerable* funds generally spoken of by the author, and particularly rated by him at 300l. a year, the annual amount received for each hospital should not be reckoned, in common, at more than 200l. And, I believe, that in this kingdom there are several county hospitals, to which the casual contributions do not exceed 100l. *per annum.*

Hence

Hence both sources of support, established and contingent, did not, until the act of last session passed, exceed 350l. a year, in a number of instances, and during a number of years.

The next mistake into which Antipeculator has fallen, is that of saying, the law ordered the hospitals to be seated "in the most central and populous town of each county." On the contrary, the law, (enacted forty years ago), neither specifies the most central, nor most populous town in each county, but fixes for the site of many of the hospitals or infirmaries, small towns, on the verge of their counties, seeming to study more the convenience of country gentlemen, meeting at the assize town, than any other consideration. Nay, some infirmaries, first stationed in tolerably central and populous towns, were afterwards removed to other towns, possessing much less of these advantages. Your correspondent appears to have a particular design in representing the seat of all our infirmaries to be fixed in populous towns; if thus seated, his project of dissolving the institutions *in toto*, would be more feasible, because in those towns the medical attendants would have a greater scope for private practice; would be more numerous, to divide the labour of visiting the infirmaries, and would be in a ratio of society from which they might derive credit and emolument, by the attention and skill shewn in their public capacity.

But, perhaps, Antipeculator does not know, that a number of the towns, in which county infirmaries are established, are far from being of the description requisite to afford those advantages. *Lifford,* in the county of Donegall, contains only 24 houses liable to the hearth-tax; *Kildare,* in the county of the same name, but 30 taxable houses; *Boyle,* in Roscommon, 66; *Wicklow,* in Wicklow county, 76; *Navan,* in Meath, 80; *Cavan,* in Cavan county, 90; and *Omagh,* in Tyrone, about an equal number. Besides ten other towns, which consist of no more than from 110 to 198 houses, in the same taxable class. Thus we have a majority * of the towns, wherein the hospitals are seated, very ill calculated to support or encourage the residence of judicious medical gentlemen, unless they were induced by a given salary; and yet the very nature of those towns, the

* There are thirty-two counties in Ireland, and an hospital in every one, excepting Leitrim, where, however, steps are now taking to have one erected.

the much greater number of whose inhabitants is poor, renders an eleemosynary asylum for their sick a laudable measure, and perfectly consistent with the object of the Legislature.

He is again inaccurate when he says, that a surgeon, instead of a physician, was appointed by law to attend our county infirmaries; for the first law expressly states, that the "sum of one hundred pounds shall be applied either to *physician or surgeon*;" and a subsequent statute is so particular as to prescribe the preparative qualification of the physician, for which it enacts, "That no person shall be appointed *Physician* to any County Infirmary, who shall not be examined and certified to be duly qualified, under the Seal of the King and Queen's College of Physicians in Ireland." It is true, a person, under the denomination of a surgeon, where one only attends, is preferable to a mere physician, for the reason that Antipeculator assigns, namely, because the latter would not be competent to perform operations, and the former should be not only qualified for the mechanical or instrumental branch, but also for the physical branch or the treatment of internal maladies. At the same time, we should recollect, that a great majority of the cases, which are admitted into county hospitals, and indeed into the generality of the hospitals, are medical cases, and require little or no operative dexterity. Let us aslo recollect the maxim of the celebrated Hunter, who pronounced "The necessity of operations is, in truth, the defect of surgery." In consonance with this sentiment the Legislature proceeded when it enacted the preceding clauses; and corresponding ideas appeared to have influenced the conduct of the Governors of the Kilkenny Hospital; (from which city Antipeculator dates his communication); for they have chosen not only a surgeon but a physician likewise to attend in that institution. Moreover, several persons who are elected under the denomination of surgeons, in different hospitals throughout the kingdom, are graduates in medicine, and are deemed so much the more eligible to have the care of the patients. No doubt the present system of medical education affords persons, brought up more particularly in the surgical line, good opportunities to understand the philosophy and principles of medicine; and consequently they might be prepared to undertake the management of internal diseases. But it is well known, that very few of those, who are intended merely for surgeons, obtain the elementary tary parts of education, which fit them to discharge with appropriate advantage the duties of a physician.

Your correspondent brings heavy charges against the county surgeons, no less than that of hiring out the spare wards of their infirmaries, and that of peculating the funds of the respective establishments; of which, he believes, that the surgeons are severally the principal administrators. That, in some cases, where the treasurer does not reside convenient to the infirmary, the money necessarily passes through the hands of the surgeon is doubtless the fact. But it does not thence follow, that the surgeon is a peculator; for all the money must first be paid into the hands of the treasurer, who issues it to the surgeon, when principal administrator, that he may apply it to the uses of the infirmary. The treasurer is always one of the most respectable gentlemen of the district, who, it is not to be supposed, would combine with the surgeon, if the latter were disposed to such baseness, for the vile purpose of defrauding the charity. Besides, the treasurer can be compelled by a summary process of law, (36th of the King) to render an exact account of the sums of money which he has received for the use of the hospital. Nay, farther, it is not to be supposed, that any surgeon, charged with such a trust, wuld be so lost to all sense of honour and probity, as to pocket the funds of a charitable institution confidentially committed to his laying out. The most irresistible motives oppose such peculation: the surgeon's education, his rank in society, his interest, all militate against the commission of an act of such depravity. And why will not Antipeculator allow to county-surgeons, in Ireland, conscience, and principle, and prudence, equal to what are found in men of a similar class in every civilized country! For my own part, I am convinced, that there exists not a surgeon of a county infirmary, in this kingdom, who pockets a single farthing of the monies set apart for the maintenance of the house.

Nor is the charge less injurious, which Antipeculator brings against the governors of those charities, who, he alleges, connive at the peculations of the surgeons, because the former are the patrons of the latter. Whereas, independent of one great bar against peculation, viz. the repute and rank of the governors, he omits observing, or is ignorant, that in many cases a number of the governors neither are patrons, nor friends, of the surgeons, being prejudiced against them by leanings to others of the profession, profession, with whom the surgeons have been, or are, competitors, &c. But, in fact, the avenues to peculation, concerning which Antipeculator is so indignant and abusive, if they ever were open, are now completely closed, by the act of the last sessions respecting our county hospitals. By that act, the treasurer of each hospital must annually verify upon oath his accounts, and each surgeon must state upon affidavit the number of patients received and relieved, before the hospital to which they belong can obtain the additional grant, 500*l. per annum.*

Antipeculator states, that in the cities of Ireland, "abundant hospitals are erected and endowed for the relief of the poor, and medical practitioners are emulous of the honour of being elected to administer their eleemosynary aid." Admitting that there are some hospitals where this species of disinterestedness may be found; it cannot be allowed that it is general, even in our first cities. In his own city, Kilkenny, whose population is rated at 14,975 persons, certain portions of the 100 l. a year are given to two medical attendants, under the denomination of physician and surgeon. In Limerick, whose inhabitants are reckoned to amount to 40,000, the surgeon of the county hospital receives a salary. In the city of Cork, estimated to contain at least 60,000 souls, there are two hospitals, the *North* and the *South* hospital, which, exclusive of the county infirmary at Mallow, receive both national and county grant, portions of which go to the payment of physicians or surgeons. And in Dublin, the metropolis of the kingdom, there are three infirmaries or hospitals, viz. the Charitable Infirmary, Mercer's Hospital, and the Hospital for Incurables, which have similar grants for similar purposes, exclusive of the one called the Meath-hospital, appointed for the county infirmary. From these instances, (the Meath-hospital excepted, whose medical attendants give up the salary to the house) we have sufficient reason to doubt that there would really be found, even in our cities and large towns, enough of medical practitioners "*emulous* of the honour of being elected to administer their eleemosynary aid;" whilst, in the small towns already mentioned, two persons rarely would be had to produce emulation, where neither honour nor emolument could be expected. The solitary instance, furnished by the County of Dublin Hospital, like every other single exception, proves the truth of the general rule; and at the same time, the hospital being seated in our chief city, whose population amounts to nearly 200,000 souls, serves to remind us, that that the necessary inducement to relinquish the salary was in full force, viz. an extensive field for practice.

With respect to the other count in the indictment, namely, that of hiring out spare wards to lodgers, it is a practice which rarely can be followed, because very few hospitals have spare wards, and the accuser himself confesses that he *heard* only of one or two instances of its being done. Indeed, his information appears to be all of the *hearsay* kind; and, what is worse, from the sounds of this *hearsay* voice does he compose the grossest imputations, uttered indiscriminately against gentlemen of a liberal profession, and who are in a respectable station in society. Thus, like the *public accuser* in revolutionary France, his charges are held in one hand, and his condemnations in the other, without having recourse to enquiry, evidence, or any other just and conscientious source of truth. If in any instance wards are let to lodgers, and only six or seven paupers on the foundation, it must be owing to a defect of contribution necessary to provide for the occupancy of all the wards, and may be ascribed to the governors and other wealthy residents in the neighbourhood, with more justice than can be converted into a pretext for censuring the surgeons. Not only in this point, but in many others where abuses have *really* crept into those establishments, I believe it will be found, upon due examination, that the cause originates in the supineness, indifference, and want of public spirit amongst those who have the power and means to act otherwise.

Gentlemen, who have the government of charitable institutions, ought to consider that, though they give *plenteously*, they do not give *enough*—money alone will not do—something more is still necessary:—they should always bear in mind, that *expenditure* is not so essential as *regulation*; money may be given with even a profuse hand; but it will be given in vain, unless they whose duty it is to direct and superintend the affairs of the institution have knowledge, attention, and perseverance. Thus qualified, they will be able to keep each part of the institution in harmony with the other, and will thereby render the whole a useful, satisfactory, and durable establishment.

But until this sense and exercise of public duty become evident, what surety would there be of a more faithful discharge of the trust by the managers of county hospitals, provided Antipeculator's proposal of dissolution were to be adopted? I cannot conceive any probable ground to expect a resuscitation of public zeal on such change, where where no new nor greater motive for it could be presented. Opening the institutions to single guinea subscribers, as he contemplates, does not appear calculated to bring about that end. It is already permitted, by law, to take place in three of our hospitals, viz. the county of Dublin, Queen's county, and Tipperary; but I do not know any practical benefit which they have reaped from that privilege. To me, considerable objections appear against it. By giving to a one guinea subscriber a privilege equal to that which is enjoyed by a three guinea subscriber, the princiciple of equity is transgressed, and gentlemen of the first rank, fortune, and liberality, are disgusted, and retire altogether from the management of the hospital, leaving it assuredly in no better, and perhaps eventually in no more numerous hands. For the guinea-men, like the voters in a borough, when they had gratified their vanity in the election of a surgeon, the motive assigned by Antipeculator, would vanish from the stage, and, true to their political exemplars, not another guinea would they give to the fire of the infirmary. What advantage, then, would be derived from numbers of this description? Is it probable that the majority of a numerous body of this nature, would answer the purpose expected from them by Antipeculator, namely, that they would surely fix on a person of probity and high professional reputation? In reality we find, that the majority of *numerous* bodies is as little to be trusted for a conscientious disposal of votes, as the majority of small bodies of men; perhaps experience would justify the conclusion, that the former is *less* to be trusted than the latter.

Besides, the whole code of infirmary laws must be repealed, and new ones framed, in order to open the doors of all the institutions to those guinea subscribers. But in fact there is no reason to make such innovation, even on the principle assumed by Antipeculator; for, by the new act already mentioned, which authorizes the establishment of dispensaries in towns and villages throughout every county, subscribers have the honour of voting for the medical officers of those establishments, and of acting in the government of all the other affairs of those supplementary hospitals. These auxiliary institutions are to be placed under the direction of the respective bodies of governors belonging to the several infirmaries; and thus at once will the utility of the infirmaries be extended, and a general relief for the sick poor, over all the counties, will be obtained. Nay, by the new act, of which Antipeculator takes no no notice, all his fuss and flourish about the sick poor are rendered null and void; for that act directs abundant "means of supplying the poor more effectually with medical assistance in the country parts of Ireland."

Before the salaries hitherto paid to the surgeons can be withdrawn, as Antipeculator advises, the existing statutes on the subject must be changed, and others made to legalize the alteration. But previous to such a *sweeping* measure, though probably in the end not a *cleansing* measure, it should be considered whether it would be wise and just to carry it into execution. We have seen, that a majority of the towns, in which county hospitals are seated, do not afford the resident surgeons opportunities for obtaining sufficient practical emolument; that these very towns require most the advantages of a local infirmary; and that a medical practitioner hardly would be found to attend *gratis* an hospital so situated. The present surgeons enjoy the salary by virtue of an Act of the Legislature, and depend on the faith of the state for a considerable part of their bread. Where, then, would be the wisdom and justice of dismissing at once the present surgeons of hospitals so circumstanced, and depriving them of their salaries? It would, surely, be not only an unwise and unjust measure, but it would also be treating the surgeons with unmerited harshness and obloquy.

If any of the surgeons are to be deprived of their salaries, let an official examination first be made into the state of all the cities and towns, in which county infirmaries are established; and having classed those cities and towns into populous and non-populous, let the surgeons of the former retire upon an annuity, and let those in the latter be continued, at least until the respective towns would arrive at the given degree of population. By this means the faith of the Legislature and State would not be broken, the objects of the charity would not be disappointed, and the desired retrenchment would eventually be obtained. Let me ask too, is it not an established rule, when a person is deprived of a salary which he had enjoyed for a length of time on the guaranty of the law, without having forfeited his right by misconduct, that he shall be entitled to an adequate compensation? For each annuitant, then, I would propose that the 100l. a year, now issued from the treasury to each hospital should be appropriated; and I would also propose, that every such annuitant should enter into engagements to contribute his services to the hospital, when required, in consultation and otherwise, otherwise, by which the advantages of his knowledge and experience would be procured in critical and intricate cases.

To the surgeons, whatever be their number, who should be continued in office, I think it would be no more than an act of common justice to allow an increase of salary, so as to make it amount to the clear sum of 200l. *per annum* for each. This is only what they are fairly entitled to, when we candidly consider their claims. A person, in order to qualify himself to be a candidate for the surgency of a county hospital in Ireland, must have received a genteel and classical education; he must have served an apprenticeship of five years to a regular bred surgeon; he must have studied at a good school of physic and surgery; he must have undergone a trying examination in the Royal College of Surgeons at Dublin; and from that College he must produce letters testimonial of his qualification to undertake the office he solicits. In the exercise of his office, he is liable to the very troublesome charge of lunatics and idiots, and on him devolves the arrangement and superintendence of the county dispensaries, over and above the care of general patients in the hospital. So that, whether on the score of information or of duty, many surgeons in the service of Government, and who have ample salaries, are not better entitled to approbation and reward than our county surgeons. To this may be added, the expensive nature of a surgeon's education, and the greatly advanced price of every necessary of life. All these circumstances put together, render unquestionable the claim of the county surgeons to an augmentation of salary; and, consequently, it cannot be supposed that the Legislature, or the constituted authorities, will think of opposing it.

Indeed, it is to be expected, that every just and unprejudiced reader will see intrinsic marks of truth in the whole of this statement, and will support the cause of a deserving class of men, who are pursued by the spirit of envy and detraction. In particular, to those of your readers, who have interest and intercourse with our senators, I would appeal in behalf of my aspersed countrymen, and intreat their services toward defeating the aims of the revilers. On the wisdom and justice of the Imperial Legislature, when suffered to have full operation, no one need be afraid to repose his trust; whilst the candour and equity of the British public ensure an impartial hearing and judgment on the occasion. In fine, gentlemen, by giving this defence immediate publicity in your Journal, and in any additional way you may think proper, you will at once serve a meritorious body of men, promote the interests of truth, and gratify, Yours, &c.

INVESTIGATOR.

*Londonderry, March* 11, 1806.

